# Physician practice in food allergy prevention in the Middle East and North Africa

**DOI:** 10.1186/s12887-017-0871-3

**Published:** 2017-05-05

**Authors:** Yvan Vandenplas, Abdulrahman Saleh AlFrayh, Bandar AlMutairi, Mahmoud Salah Elhalik, Robin J. Green, Joseph Haddad, Emad Abdulqader Koshak, Mohamad Miqdady, Nezha Mouane, Mohamed Salah, Gamal Samy, Marzieh Tavakol, Andrea von Berg, Hania Szajewska

**Affiliations:** 1 Department of Pediatrics, UZ Brussel, Vrije Universiteit Brussel, Brussels, Belgium; 2 Department of Pediatrics, King Saud University College Of Medicine, Riyadh, Kingdom of Saudi Arabia; 3 Bandar Medical Center, Jahra City, Kuwait; 4 Department of Pediatrics and Neonatology, Latifa Hospital, Dubai Health Authority, Dubai, UAE; 5 Department of Paediatrics and Child Health, University of Pretoria, Pretoria, South Africa; 6 Department of Pediatrics, Saint George University Hospital, Balamand University, Beirut, Lebanon; 7 Department of Internal Medicine, King Abdulaziz University Hospital, Jeddah, Kingdom of Saudi Arabia; 8 Pediatric Gastroenterology, Hepatology & Nutrition Division, Sheikh Khalifa Medical City, Abu Dhabi, United Arab Emirates; 9 Gastroenterology Nutrition Department, Children Hospital Ibn Sina, University Mohammed V Faculty of Medicine, Rabat, Morocco; 10 Nestlé Nutrition, Dubai, United Arab Emirates; 11 Department of Medical Childhood Studies, Institute of Postgraduate Childhood Studies, Ain Shams University, Cairo, Egypt; 12 Department of Allergy and Clinical Immunology, Shahid Bahonar Hospital, Alborz University of Medical Sciences, Karaj, Iran; 13 Department of Paediatrics, Research Institute, Marien-Hospital-Wesel, Wesel, Germany; 14 The Medical University of Warsaw, Warsaw, Poland

**Keywords:** Allergy, atopy, prevention, breastfeeding, hydrolysed formula, complementary feeding

## Abstract

**Background:**

A number of scientific organisations have developed guidelines for the primary prevention of allergic disease through nutritional interventions. However, even if the best evidence-based guidelines are available, these guidelines do not necessarily lead to adherence and improved health outcomes.

**Method:**

To determine how closely the practice of physicians in select Middle Eastern and North African countries compares with the current recommendations on the primary prevention of allergy a survey study was performed using a structured questionnaire and convenience sampling.

**Results:**

A total of 1481 physicians responded, of which 66.1% were pediatricians. A total of 76.6% of responding physicians routinely identify infants who are at risk for developing allergy. In infants at risk for developing allergy, 89.1% recommend exclusive breastfeeding for at least 4 months. In contrast to current recommendations, 51.6% routinely recommend avoidance of any allergenic food in the lactating mother. In infants at risk of developing allergy who are completely formula fed, standard infant formula was recommended by 22.5% of responders. Of the responding physicians, 50.6% would recommend delaying the introduction of complementary food in infants at risk of allergy compared to those not at risk, whereas 62.5% would recommend postponing the introduction of potentially allergenic foods. Only 6.6% stated they follow all current recommendations on food allergy prevention.

**Conclusion:**

The results of this survey suggest that a substantial part of responding physicians from select Middle Eastern and North African (MENA) countries do not follow current recommendations on primary prevention of allergic disease through nutritional interventions.

**Electronic supplementary material:**

The online version of this article (doi:10.1186/s12887-017-0871-3) contains supplementary material, which is available to authorized users.

## Background

The rising number of children and adults with allergic disorders such as asthma, allergic rhinitis, atopic dermatitis and food allergy worldwide is a major public health concern [1]. The origins of this increase are still not well understood. However, it has been hypothesised that factors such as the mode of delivery (vaginal vs caesarean), aberrant gut microbiota, use of antibiotics in the early neonatal period, mode of feeding (breast vs formula feedings) and mode of weaning (early vs later) contribute to the development of allergic diseases. If so, the prevention of allergic disorders through modification of early nutritional interventions is of great interest.

A number of scientific organisations have developed guidelines for the primary prevention of allergic disease through nutritional interventions [2–5]. Maternal avoidance diets that avoid foods such as milk and eggs during pregnancy and lactation were previously recommended [6], but this is not the recommendation of recent guidelines [2–5]. For all infants, exclusive breastfeeding for up to 6 months is a desirable goal [3]. Infants with a documented hereditary risk of allergy (i.e., an affected parent and/or sibling), who cannot be breastfed exclusively, should receive either a partially or extensively hydrolysed formula as a means of preventing allergic reactions. There is no convincing scientific evidence that avoidance or delayed introduction of potentially allergenic foods beyond 4–6 months reduces allergies in infants considered to have an increased risk for developing allergic diseases or in those not considered to have an increased risk.

Even if the best evidence-based guidelines are available, they do not necessarily lead to improved health outcomes. Hence, there is an interest in knowledge translation. A number of barriers between guidelines (or other research) and health outcomes exist. They extend from awareness to adherence [7]. Overall, we have limited knowledge on the practices provided to patients for the primary prevention of allergic diseases. The purpose of this survey study was to determine how closely the practice of physicians in select MENA countries compare with current recommendations on primary prevention of allergy.

## Methods

The questionnaire was developed by a working group of experts in the field of allergy prevention and members of the target population with reference to existing research literature. The working group comprised regional experts from representative countries across the MENA region including Egypt, Iran, Kingdom of Saudi Arabia, Kuwait, Lebanon, Morocco and the United Arab Emirates. These regional experts worked in consultation with international experts from Belgium, Germany, Poland and South Africa. It was also developed in collaboration with Nestlé Nutrition Institute Middle East during a meeting in Dubai in January 2015.

The questionnaire consisted of two parts. The first part focused on responders’ characteristics. The second part of the questionnaire included specific closed questions (i.e. response options were available) with regard to an infant at high-risk of allergy (Additional file 1). This has been defined as an infant without allergic symptoms but with at least one first-degree relative (parent or sibling) having one or more allergic disease (i.e. allergic rhinitis, asthma, urticaria, atopic dermatitis [eczema], food allergy) [4]. The allergic disease should have been diagnosed by a physician or the patient must be clearly dependant on a relevant treatment. The language of the questionnaire was in English or French (in Algeria and Morocco). Institutional review board approval was obtained from each participating centre prior to study commencement.

The survey assessed adherence to practice recommended by international guidelines which can be summarised that [3, 4, 8, 9]:

- infants should be assessed for allergy risk
- infants should be exclusively breastfed for at least 4 months
- maternal diet of lactating mothers should not avoid potential allergens
- formula-fed infants at risk of developing allergy should be fed a formula clinically proven to have reduced allergenicity
- infants at risk of allergy should not have delayed introduction of complementary feeding or potentially allergenic complementary foods

Convenience sampling was employed to collect data. Surveys were distributed at national and regional paediatric meetings in the MENA region, including ELITE Conference, Lebanese Pediatric Society Meeting, UMEMPS Meeting, Mediterranean Congress of Pediatrics, Egyptian Society for Neonatal and Preterm Care Meeting, and Arab Neonatal Conference. Surveys were completed anonymously. Descriptive statistics were used to summarise the demographics of the respondents and their responses to questions.

## Results

Two thousand surveys were distributed. A total of 1572 surveys were completed, with 19 surveys excluded due to allergy practice not being adequately completed and an additional 72 excluded because they were from unspecified or non-relevant healthcare specialities. Therefore, the total number of responders was 1481 (Additional file 2). Of these surveys, 1100 were completed at national meetings, and 381 were completed at regional meetings. The characteristics of participating physicians are presented in Table 1 **.** The majority of participants were below 40 years of age and only 3.3% of respondents were over 60 years of age. The male to female ratio was approximately 1:1 (53.9% vs 46.1%, respectively). Within the sample, 979 (66.1%) were paediatricians, 341 (23.0%) were general physicians, 105 (7.1%) were paediatric gastroenterologists and 56 (3.8%) were classified as others. The majority of participants, 1048 (70.8%), reported working within the government facility, 422 (28.5%) reported working in a private facility and 11 (0.7%) reported working in other practices. Most participants 1294 (90.7%) worked full time.

**Table 1:** The demographic and self-reported characteristics of the survey respondents (*N* = 1481)

Demographic	Category	Number	Percent
Specialty (*N* = 1481)	Paediatricians	979	66.1
	General physicians	341	23.0
	Paediatric gastroenterologists	105	7.1
	Others	56	3.8
Practice type (*N* = 1481)	Governmental	1048	70.8
	Private	422	28.5
	Others	11	0.7
Practice location (*N* = 1471)	Hospital only	957	65.1
	Clinic only	447	30.4
	Others	67	4.6
Work status (*N* = 1427)	Full time	1294	90.7
	Part time	133	9.3
Country (*N* = 1448)	Saudi Arabia	521	36.0
	Morocco	380	26.2
	Lebanon	136	9.4
	Algeria	116	8.0
	Egypt	84	5.8
	Iran	69	4.8
	Iraq	43	3.0
	Kuwait	38	2.6
	Others	61	4.2
Gender (*N* = 1466)	Male	790	53.9
	Female	676	46.1
Age group (*N* = 1467)	<40 years	597	40.7
	40–50 years	504	34.4
	50–60 years	317	21.6
	>60 year	49	3.3

The most represented countries were Saudi Arabia (36.0%), Morocco (26.2%), Lebanon (9.4%), Algeria (8.0%), Egypt (5.8%), Iran (4.8%), Iraq (3.0%) and Kuwait (2.6%). For the remaining countries, the response was below 1% (Table 1).

A total of 76.6% of responding physicians routinely identify infants at risk for developing allergy and 89.1% recommend exclusive breastfeeding for at least 4 months. In addition, 51.6% routinely recommend avoidance of any allergenic food in the lactating mother. In an infant at risk of developing allergy who is both breast and formula fed (mixed feeding), 25.0% of physicians would recommend standard infant formula, whereas 69% would recommend a partial or extensive hydrolysate formula. In an at-risk infant who is completely formula fed, 22.5% of physicians would recommend standard infant formula, whereas 71.1% would recommend a partial or extensive hydrolysate formula. Of the responding physicians, 50.6% would recommend delaying the introduction of complementary food and 62.5% would recommend postponing the introduction of potentially allergenic foods. Only 6.6% of respondents self-reported following all current recommendations on the primary prevention of allergy as per the recommendations summarised in the methodology (Table 2). There were no meaningful differences between specialities, with only 6.1% of paediatricians, and 7.6% of pediatric gastroenterologists self-reporting that they follow all current recommendations.

**Table 2:** Questionnaire results

Question	All physicians				Pediatricians				Pediatric Gastroenterologists			
	N	n		%	N	n		%	N	n		%
Do you routinely identify infants at risk for developing allergy?	1476	Yes	1130	76.6	975	Yes	747	76.6	105	Yes	76	72.4
		No	346	23.4		No	228	23.4		No	29	27.6
In an infant at risk for developing allergy, do you recommend exclusive breastfeeding for at least 4 months?	1471	Yes	1310	89.1	972	Yes	859	88.4	105	Yes	95	90.5
		No	161	10.9		No	113	11.6		No	10	9.5
In an infant at risk for developing allergy, who is exclusively breast fed, do you routinely recommend avoidance of any of the allergenic foods (cow’s milk, egg, fish, peanut, soy, wheat) in the lactating mother?	1462	Yes	754	51.6	965	Yes	548	56.8	105	Yes	33	31.4
		No	708	48.4		No	417	43.2		No	72	68.6
In an infant at risk for developing allergy, who is breast and formula fed (mix feeding), which type of formula do you recommend?												
• Standard (regular) infant formula	1458	365		25.0	961	211		22.0	105	33		31.4
• pH formula (HA formula)		890		61.0		605		63.0		58		55.2
• Extensively hydrolysed formula		117		8.0		87		9.1		10		9.5
• Soy infant formula		57		3.9		42		4.4		2		1.9
• Other		23		1.6		15		1.6		2		1.9
• Combination of formula		6		0.4		1		0.1		0		0
In an infant at risk for developing allergy, who is exclusively formula, which type of formula do you recommend?												
• Standard (regular) infant formula	1424	320		22.5	939	183		19.5	104	38		36.5
• pH formula (HA formula)		822		57.7		533		56.8		55		52.9
• Extensively hydrolysed formula		191		13.4		148		15.8		10		9.6
• Soy infant formula		73		5.1		61		6.5		0		0
• Other		14		1.0		10		1.1		1		1.0
• Combination of formula		4		0.3		4		0.4		0		0
In an infant at risk for developing allergy, do you recommend delaying the introduction of complementary foods compared with non-at-risk infants?	1444	Yes	731	50.6	950	Yes	495	52.1	104	Yes	53	51.0
		No	713	49.4		No	455	47.9		No	51	49.0
In an infant at risk for developing allergy, do you recommend postponing the introduction of potentially allergenic foods (egg, cow’s milk, wheat, fish, peanut) compared with non-at-risk infants?	1434	Yes	896	62.5	942	Yes	576	61.1	103	Yes	65	63.1
		No	538	37.5		No	366	38.9		No	38	36.9
Follow all current recommendations on primary prevention of allergy	1481	Yes	98	6.6	979	Yes	60	6.1	105	Yes	8	7.6
		No	1383	93.4		No	919	93.9		No	97	92.4

*HA* hypo-allergenic, *pH* partially hydrolysed

## Discussion

### Principal findings

The survey responses suggest that the majority of participating physicians from these select MENA countries routinely identify infants at risk for developing allergy. The recommendation for allergy prevention that is most commonly followed was exclusive breastfeeding for at least 4 months. Contrary to current recommendations, avoidance of any allergenic food in the lactating mother is often recommended, and a substantial number of physicians appear to still recommend standard infant formula in infants who are both breast and formula fed or who are exclusively formula fed. These data suggest a large number of physicians are following outdated recommendations, such as the 2000 American Academy of Pediatrics guidelines, which advise maternal avoidance diets for prevention. [6] These guidelines have been superseded by guidelines that no longer make this recommendation. [3, 4] Over 50% of physicians would recommend delaying the introduction of complementary food, and over 60% would recommend postponing the introduction of potentially allergenic foods. Current guidelines encourage timely introduction of complementary food [3, 4]. Only 6.6% of responders self-reported following all current recommendations on the primary prevention of allergy.

### Strengths and weaknesses of the study

This survey provides an up-to-date overview on the current state of allergy prevention practice in select MENA countries. However, the sample sizes for many of these countries were not representative.

The survey method was not entirely robust. The study is limited by its convenience sample and its inability to generalise to participants other than those studied. Practitioners who provided our data were not selected through a randomisation process. We also accept that there remains the potential for a response bias because those who do not advise on allergy prevention may be less likely to complete the survey. Additionally no steps were taken to ensure that a physician was not able to take the survey twice.

The survey documented only the physicians’ self-reported management of an infant at risk for developing an allergy. However, we did not investigate actual practice in the countries involved. Furthermore, we realise that because of the different healthcare systems in many countries, the nature and role of each type of physician may vary considerably. Access to specialised formulas may also differ depending on the country and healthcare system.

The analysis of the questionnaires does not allow us to conclude on the reasons for failure to comply with recommendations, and therefore does not suggest specific interventions. One explanation for the lack of knowledge of respondents may have been that some of these were physicians who were usually not expected to provide allergy prevention advice.

### Comparisons with other studies

One study from Brazil on the knowledge and practice of physicians and nutritionists regarding the prevention of food allergy has shown similar results [10]. Only some of the recommendations issued by scientific organisations were followed in practice. Most of the discrepancies between recommendations and practice, as with the present study, were in the timings of the introduction of complementary feeding and the avoidance of potentially allergenic foods. The study concluded that there are gaps in knowledge among healthcare providers regarding the prevention of food allergy. A recent French study confirmed these gaps, as hydrolysates are not prescribed in all units [11].

## Conclusions

We have provided an important, albeit preliminary, insight into the routine provision of primary prevention of allergy advice within select MENA countries. The results of our survey suggest that a substantial part of responding physicians from this region do not follow current recommendations on allergy prevention in infants at risk of allergy. The findings of this survey may suggest that more educational efforts are required to increase the awareness and/or the adherence to current guidelines.

## Additional files

Additional file 1: Survey questionnaire in English. (PDF 23 kb)

Additional file 2: Survey data tables. (PDF 41 kb)

## Abbreviations

HA: Hypo-allergenic
MENA: Middle East and North Africa
pH: Partially hydrolysed
