# Supplementary material for: Physician practice in food allergy prevention in the Middle East and North Africa
Source: BMC Pediatr. 2017 May 5;17:118. doi: 10.1186/s12887-017-0871-3 (PMC5418846; doi:10.1186/s12887-017-0871-3)
Supplement: Supplementary file 1 — Survey questionnaire in English. (PDF 23 kb) [file 12887_2017_871_MOESM1_ESM.pdf]

Allergy Prevention Survey

For the purpose of this survey, an **infant at risk for developing allergy is** defined as an infant without allergic symptoms but with at least one first degree relative (parent or sibling) with one or more allergic disease, including : allergic rhinitis, asthma, urticaria, atopic dermatitis (eczema), food allergy. The allergic disease must by diagnosed by a physician or the patient must clearly depend on a relevant treatment.

1) Do you routinely identify **infants at risk for developing allergy**?

    a) Yes

    b) No

2) In the **infant at risk for developing allergy**, do you recommend exclusive breastfeeding for at least 4 months?

    a) Yes

    b) No

3) In the **infant at risk for developing allergy**, who is exclusively breast fed; do you routinely recommend avoidance of any of the allergenic foods (cow's milk, egg, fish, peanut, soy, wheat) in the lactating mother?

        i) Yes

If yes: which foods? ……………………………………………………………………………………

    b) No

4) In the **infant at risk for developing allergy**, who is breast and formula fed (mix feeding); which type of formula do you recommend?

    a) Standard (Regular) infant formula

    b) Partially hydrolysed formula (HA formula)

    c) Extensive hydrolysed formula

    d) Soy infant formula

    e) Other? Please specify…………….

5) In the **infant at risk for developing allergy**, who is completely formula fed; which type of formula do you recommend?

    a) Standard (Regular) infant formula

b) Partially hydrolysed formula (HA formula)

c) Extensive hydrolysed formula

d) Soy infant formula

e) Other? Please specify…………….

6) In the **infant at risk for developing allergy**, do you recommend delaying the introduction of complementary foods compared to non-at-risk infants?

a) Yes

b) No

7) In the **infant at risk for developing allergy**, do you routinely recommend postponing the introduction of potentially allergenic foods (egg, cow's milk, wheat, fish, peanut) compared to non-at-risk infants?

a) Yes

i) If yes: which foods? ………………………………………………………………………………………

b) No
