# Supplementary material for: Physician practice in food allergy prevention in the Middle East and North Africa
Source: BMC Pediatr. 2017 May 5;17:118. doi: 10.1186/s12887-017-0871-3 (PMC5418846; doi:10.1186/s12887-017-0871-3)
Supplement: Supplementary file 2 — Survey data tables. (PDF 41 kb) [file 12887_2017_871_MOESM2_ESM.pdf]

1. General Data (N=1481)

1.1A Specialty

	N	%
General physician	341	23.0
Pediatrics	979	66.1
Pediatric Gastroenterologist	105	7.1
Other	56	3.8
Total	1481	100.0

1. General Data (N=1481)

1.2 Practice type

	N	%
Government facility	1048	70.8
Private facility	422	28.5
Other or missing	11	0.7
Total	1481	100.0

1. General Data (N=1481)

1.3 Practice location

	N	%
Clinic	447	30.2
Hospital	967	65.3
Others or missing	67	4.5
Total	1481	100.0

1. General Data (N=1481)

1.4 Country

	N	%
ALGERIA	116	8.0
BAHRAIN	1	0.1
CHILE	1	0.1
EGYPT	84	5.8
ENGLAND	1	0.1
GAZA	1	0.1
IRAN	69	4.8
IRAQ	43	3.0
JORDAN	7	0.5
KUWAIT	38	2.6
LEBANON	136	9.4
MOROCCO	380	26.2
OMAN	5	0.3
PAKISTAN	3	0.2
PALESTINE	2	0.1
SAUDI ARABIA	521	36.0
SOUTH KOREA	1	0.1
SUDAN	3	0.2
SYRIA	13	0.9
TURKEY	1	0.1
UAE	16	1.1
UKRAINE	1	0.1
USA	4	0.3
YEMEN	1	0.1
Total	1448	100.0

1. General Data (N=1481)

1.5 Gender

	N	%
Male	790	53.9
Female	676	46.1
Total	1466	100.0

1. General Data (N=1481)

1.6 Age group

	N	%
< 40 years	597	40.7
40-50 years	504	34.4
50-60 years	317	21.6
> 60 years	49	3.3
Total	1467	100.0

1. General Data (N=1481)
1.7 Work status

	N	%
Full time	1294	90.7
Part time	133	9.3
Total	1427	100.0

2. Allergy Data (N=1481)
2.1 Infants at risk for developing allergy identified
2.1A All Subjects

	N	%
Yes	1130	76.6
No	346	23.4
Total	1476	100.0

2. Allergy Data (N=1481)
2.2 Exclusive breastfeeding for at least 4 months
2.2A All Subjects

	N	%
Yes	1310	89.1
No	161	10.9
Total	1471	100.0

2. Allergy Data (N=1481)

2.3 Breastfeeding - Avoiding allergenic foods

2.3A All Subjects

	N	%
Yes	754	51.6
No	708	48.4
Total	1462	100.0

2. Allergy Data (N=1481)

2.4 Mixed feeding - Formula type

2.4A All Subjects

	N	%
Standard (Regular) infant formula	365	25.0
Partially hydrolysed formula (HA formula)	890	61.0
Extensive hydrolysed formula	117	8.0
Soy infant formula	57	3.9
Other formula	23	1.6
Combination of formulae	6	0.4
Total	1458	100.0

2. Allergy Data (N=1481)
2.5 Exclusive formula feeding - Formula type
2.5A All Subjects

	N	%
Standard (Regular) infant formula	320	22.5
Partially hydrolysed formula (HA formula)	822	57.7
Extensive hydrolysed formula	191	13.4
Soy infant formula	73	5.1
Other formula	14	1.0
Combination of formulae	4	0.3
Total	1424	100.0

2. Allergy Data (N=1481)

2.6 Delaying introduction of complementary foods

2.6A All Subjects

	N	%
Yes	731	50.6
No	713	49.4
Total	1444	100.0

2. Allergy Data (N=1481)

2.7 Postponing introduction of potentially allergenic foods

2.7A All Subjects

	N	%
Yes	896	62.5
No	538	37.5
Total	1434	100.0

2. Allergy Data (N=1481)
2.8 Score of 100% of the right answers
2.8A All Subjects

	N	%
Yes	98	6.6
No	1383	93.4
Total	1481	100.0

2. Allergy Data - Pediatrics (N=979)

2.1 Infants at risk for developing allergy identified

2.1A All Subjects

	N	%
Yes	747	76.6
No	228	23.4
Total	975	100.0

2. Allergy Data - Pediatrics (N=979)

2.2 Exclusive breastfeeding for at least 4 months

2.2A All Subjects

	N	%
Yes	859	88.4
No	113	11.6
Total	972	100.0

2. Allergy Data - Pediatrics (N=979)
2.3 Breastfeeding - Avoiding allergenic foods
2.3A All Subjects

	N	%
Yes	548	56.8
No	417	43.2
Total	965	100.0

2. Allergy Data - Pediatrics (N=979)
2.4 Mixed feeding - Formula type
2.4A All Subjects

	N	%
Standard (Regular) infant formula	211	22.0
Partially hydrolysed formula (HA formula)	605	63.0
Extensive hydrolysed formula	87	9.1
Soy infant formula	42	4.4
Other formula	15	1.6
Combination of formulae	1	0.1
Total	961	100.0

2. Allergy Data - Pediatrics (N=979)
2.5 Exclusive formula feeding - Formula type
2.5A All Subjects

	N	%
Standard (Regular) infant formula	183	19.5
Partially hydrolysed formula (HA formula)	533	56.8
Extensive hydrolysed formula	148	15.8
Soy infant formula	61	6.5
Other formula	10	1.1
Combination of formulae	4	0.4
Total	939	100.0

2. Allergy Data - Pediatrics (N=979)

2.6 Delaying introduction of complementary foods

2.6A All Subjects

	N	%
Yes	495	52.1
No	455	47.9
Total	950	100.0

2. Allergy Data - Pediatrics (N=979)

2.7 Postponing introduction of potentially allergenic foods

2.7A All Subjects

	N	%
Yes	576	61.1
No	366	38.9
Total	942	100.0

2. Allergy Data - Pediatrics (N=979)
2.8 Score of 100% of the right answers
2.8A All Subjects

	N	%
Yes	60	6.1
No	919	93.9
Total	979	100.0

2. Allergy Data - Pediatric Gastroenterologists (N=105)
2.1 Infants at risk for developing allergy identified
2.1A All Subjects

	N	%
Yes	76	72.4
No	29	27.6
Total	105	100.0

2. Allergy Data - Pediatric Gastroenterologists (N=105)
2.2 Exclusive breastfeeding for at least 4 months
2.2A All Subjects

	N	%
Yes	95	90.5
No	10	9.5
Total	105	100.0

2. Allergy Data - Pediatric Gastroenterologists (N=105)
2.3 Breastfeeding - Avoiding allergenic foods
2.3A All Subjects

	N	%
Yes	33	31.4
No	72	68.6
Total	105	100.0

2. Allergy Data - Pediatric Gastroenterologists (N=105)
2.4 Mixed feeding - Formula type
2.4A All Subjects

	N	%
Standard (Regular) infant formula	33	31.4
Partially hydrolysed formula (HA formula)	58	55.2
Extensive hydrolysed formula	10	9.5
Soy infant formula	2	1.9
Other formula	2	1.9
Total	105	100.0

2. Allergy Data - Pediatric Gastroenterologists (N=105)
2.5 Exclusive formula feeding - Formula type
2.5A All Subjects

	N	%
Standard (Regular) infant formula	38	36.5
Partially hydrolysed formula (HA formula)	55	52.9
Extensive hydrolysed formula	10	9.6
Other formula	1	1.0
Total	104	100.0

2. Allergy Data - Pediatric Gastroenterologists (N=105)
2.6 Delaying introduction of complementary foods
2.6A All Subjects

	N	%
Yes	53	51.0
No	51	49.0
Total	104	100.0

2. Allergy Data - Pediatric Gastroenterologists (N=105)
2.7 Postponing introduction of potentially allergenic foods
2.7A All Subjects

	N	%
Yes	65	63.1
No	38	36.9
Total	103	100.0

2. Allergy Data - Pediatric Gastroenterologists (N=105)
2.8 Score of 100% of the right answers
2.8A All Subjects

	N	%
Yes	8	7.6
No	97	92.4
Total	105	100.0
